# Double Robertsonian translocations in an infertile patient with macrocytic anemia: a case report

**DOI:** 10.1186/s13039-020-00482-6

**Published:** 2020-04-16

**Authors:** Ramakrishnan Sasi, Jamie Senft, Michelle Spruill, Soham Rej, Peter L. Perrotta

**Affiliations:** 1 Department of Pathology, Anatomy and Laboratory Medicine, West Virginia University, Health Sciences Center, Morgantown, WV 26506 USA; 2 Jewish General Hospital/Lady Davis Institute, Montreal, Quebec Canada

**Keywords:** Double Robertsonian translocations, Infertility, Ribosomopathies, Macrocytic anemia

## Abstract

**Background:**

Constitutional heterologous double Robertsonian translocations (DRT) between chromosomes 13/14 and chromosomes 14/15 with 44 chromosomes are extremely rare. In this case report, we present the karyotype analysis of metaphases prepared from bone marrow, peripheral blood and cultured skin tissue cells. These showed only 44 chromosomes with DRT involving chromosomes 13, 14 and 15. To our knowledge this is the first reported case with DRT involving chromosomes 14 and 15.

**Case presentation:**

The patient is an 81-year-old infertile male with a history of persistent macrocytic anemia (MA). The patient presented with fatigue, paleness of the skin, shortness of breath, lightheadedness and occasional dizziness. Work-up for common causes of macrocytic anemias in this case were excluded: folate/vitamin B12 deficiency, hypothyroidism, liver diseases, hemolysis, bleeding, alcoholism, exposure, HIV infection, chemotherapy or blood loss, drug-toxicity effect, or myelodysplasia. This individual with DRT had only six nucleolus organizer regions (NORs), instead of the usual ten, of which 50% of the 6 NORs were inactive (*n* = 3).

**Conclusion:**

In this case, macrocytic anemia (MA) appeared to be due to reduction in active NORs in DRT. We postulate that the marked reduction in active NORs leads to reduction in active nucleoli formation, which may be limiting ribosomal RNA synthesis, contributing to MA. It is probable that reduction in NOR activity affected normal DNA synthesis and cellular functions.

## Background

Macrocytic anemia is caused by impaired DNA synthesis [1, 2]. Reduction in the active number of NORs in this patient may be the cause of macrocytic anemia. We hypothesize that the DRT may be responsible for the MA due to fewer NORs and subsequent reduced rRNA activity. This is the first case of a macrocytic anemia patient with two heterologous RT involving chromosomes 13 and 14 and chromosomes 14 and 15.

A Robertsonian Translocation (RT) is the fusion of two acrocentric chromosomes. The first RT was described by American insect cytogeneticist W.R.B. Robertson in 1916 in grasshoppers. RT is the most common structural chromosome aberration in humans (1/1000 live births). Only 3% of RT are acquired; 97% are constitutional [1–3].

Approximately 95% of RTs are formed between two heterologous acrocentric chromosomes (13, 14, 15, 21 and 22) to form a single submetacentric chromosome [3]. The majority of RTs are between either chromosomes 13 and 14 (75%), or chromosomes 14 and 21(8%) [3]. RTs between chromosomes 14 and 15 are extremely rare. Heterozygous RTs can be transmitted through many generations, but homozygous RTs always occur de novo*.* RT formation involves the simultaneous loss of both short arms resulting in the loss of multiple copies of rRNA genes but, because these genes are redundant, this loss is not thought to produce any deleterious outcome.

Constitutional double heterozygous RTs with 44 chromosomes are very rare. Cases are reported between unions of consanguineous parents that are RT heterozygotes [3, 4]. To the best of our knowledge, no cases with two heterologous RTs between chromosomes 13 and 14 and chromosomes 14 and 15 with 44 chromosomes have been reported. RTs may be congenital or acquired and have been associated with various premalignant and malignant hematological disorders. However, more than 90% of RT formations are constitutional [3].

Macrocytic anemias (MA) are a group of anemias in which the circulating erythrocytes are larger than normal with high MCV [1, 2]. MA may be associated with a variety of non-neoplastic conditions, such as vitamin B-12 and folate deficiencies, alcoholism, liver diseases, hypothyroidism, gastric diseases and gastrectomy, parvovirus infection, chemotherapy, and drug/toxin-induced disorders of DNA synthesis and replication [1, 2]. MA is also observed in various neoplastic conditions such as myelodysplastic syndrome (MDS), erythroleukemia and aplastic anemia [5, 6]. In some of the above conditions, macrocytosis is associated with abnormal nucleotide metabolism and/or defects in DNA polymerization. In this paper, we present a case of persistent MA in an adult patient with two constitutional heterologous RTs.

## Case presentation

The patient is an 81-year-old sterile, non-consanguineous Caucasian male who is a retired army engineer with a history of macrocytic anemia. The patient also had fatigue, paleness of the skin, shortness of breath, lightheadedness and occasional dizziness. The base line hemoglobin was 10.5 g/dl. After being placed on Vitamin B12 supplementation, hemoglobin increased and MCV decreased, but neither returned to normal range. To rule out MDS, a bone marrow biopsy was performed.

**Fig. 1:**
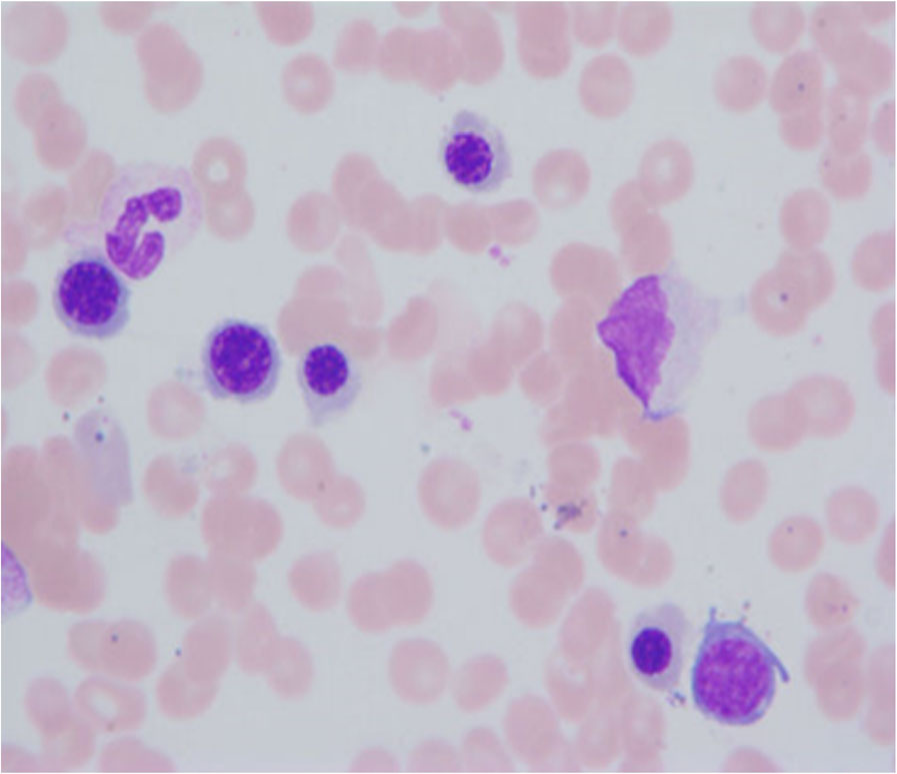
Bone Marrow aspirate smears, Wright-Giemsa Stain 1000X

**Fig. 2:**
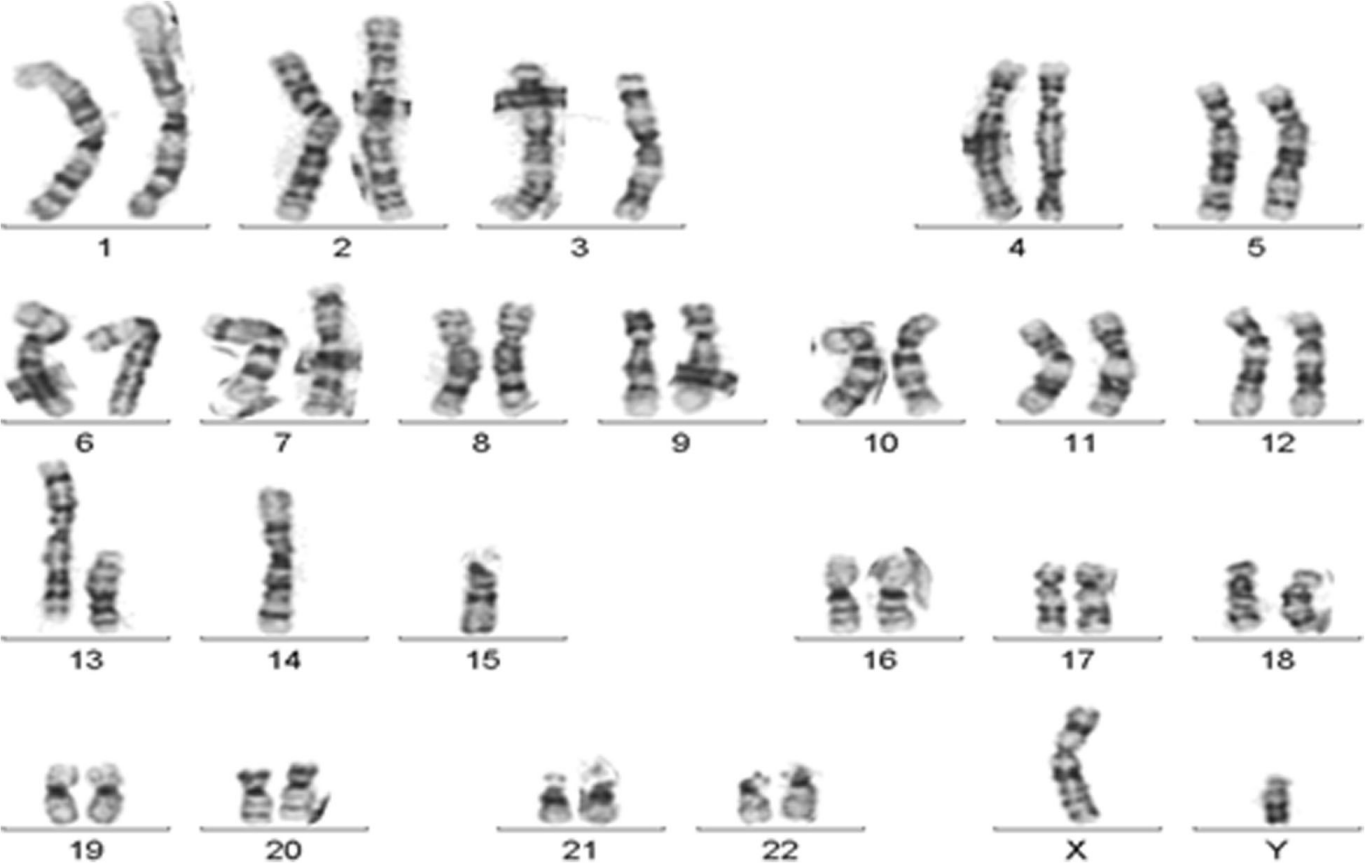
G-banded metaphase chromosome preparations from unstimulated overnight bone marrow culture. All 20 chromosomes analyzed exhibited 44 chromosomes and no normal chromosome 14. Karyotype: 44,XY,rob(13;14) (q10,q10), rob(14;15) (q10;q10) [20] . The same heterologous RT formation was observed both in peripheral blood and epithelial tissue derived metaphase preparations. These karyotype results from multiple tissue types indicate the constitutional nature of the double RTs

BM morphometric and flow cytometric analysis revealed proportionally normal myeloid, monocytic and lymphoid elements with no increased blasts, plasma cells and no aberrant myeloid or lymphoid maturation (Fig. 1). Both conventional and FISH metaphase analysis proved the presence of 44 chromosomes containing (13;14) and (14;15) Robertsonian translocations (Figs. 2 and 4). Figure 2 shows the ISCN karyotype: 44,XY,rob(13;14)(q10;q10),rob(14;15)(q10;q10). No normal chromosome 14 was present in the karyotype.

**Fig. 3:**
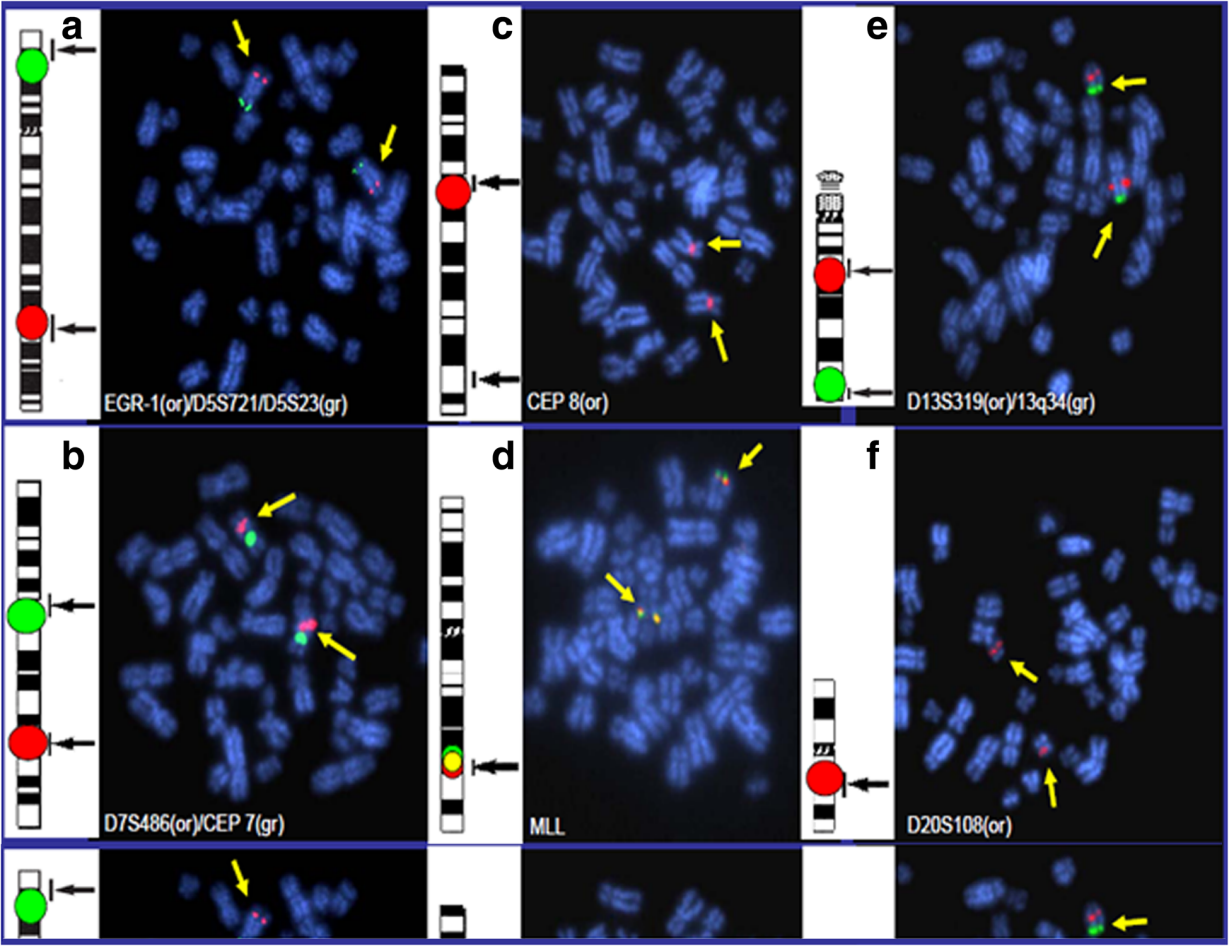
FISH studies for MDS. BM metaphase spreads were hybridized with fluorescent DNA probes for: **a**: 5q31(EGR1 orange), 5p15.2(D5S721 green), **b**: 7q31(D7S486 orange), CEP 7(alpha satellite green) **c**: CEP 8(alpha satellite orange), **d**: 11q23(5’MLL green,3’MLL orange), **e**: 13q14.3(D13S319 orange), 13q34(green), **f**: 20q12 (D20S108 orange) No MDS specific FISH aberrations were observed

**Fig. 4:**
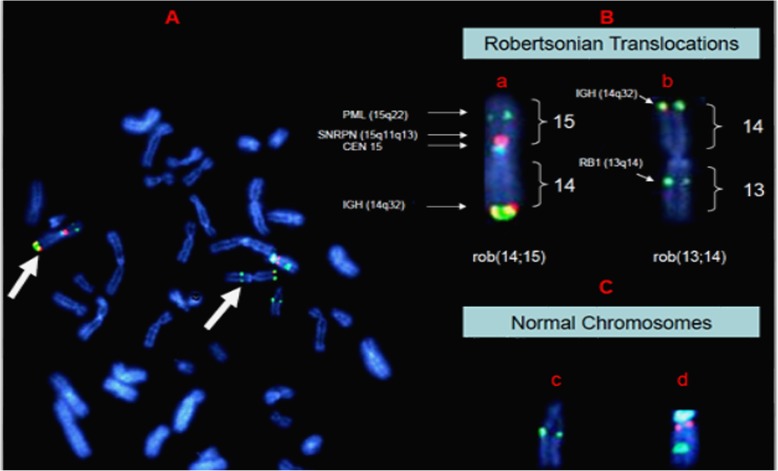
FISH characterization of double heterologous Robertsonian translocations. Bone marrow metaphase hybridized with fluorescent DNA probes for 15q22 (PML green), 15q11.2 (SNRPN orange), Cen15 (alpha satellite aqua), chromosome 14q32 (5’IGH green, 3’IGH orange) and chromosome 13q14(RB-1 green). Note the absence of a normal chromosome 14

No MDS/AML specific chromosome aberration was observed in the karyotype analysis or interphase and metaphase MDS FISH panel studies (Figs. 2 and 3). A double heterozygous RT consisting of rob(13q;14q) and rob (14q;15q) was further confirmed by metaphase FISH analysis using multiple FISH probes encompassing various regions of chromosome 13, 14 and 15 (Fig. 4). Both RTs were present in 100% of bone marrow, peripheral blood, and cultured fibroblast cells; therefore they were found to be constitutional and not mosaic in nature.

Bone marrow aspirate smears from the patient were stained with Wright- Giemsa Stain 1000X (Fig. 1). The patient’s blood cells are larger than normal, with nuclei that appear to be immature relative to cellular cytoplasm. The five-color flow cytometric assay utilized the Cytomics FC500 with CXP software and fluorescent monoclonal antibodies (data not shown). Conventional G-banded metaphase chromosome analysis was performed on an unstimulated overnight bone marrow culture, a 72 h PHA-stimulated peripheral blood culture and bone marrow fibroblast cultures (Fig. 2). Bone marrow fibroblast cells were obtained by repeated culturing of bone marrow samples in a T25 flask for 3–4 weeks with weekly replacement of fresh media. Fibroblast cells were incubated overnight with Colcemid and metaphase chromosomes were prepared for analysis by standard methods. FISH analysis was performed on bone marrow metaphase chromosomes preparations. All fluorescently labeled DNA probes were purchased from Abbott Molecular and hybridizations done according to the manufacturer’s protocol.

In humans, the NORs are located on the secondary constrictions of the five pairs of acrocentric chromosomes. Active NORs can be stained by silver nitrate and visualized with the use of the silver staining technique. This technique selectively stains active NORs but does not detect the presence of rRNA genes. To determine the number of active nucleolar organizing regions (NORs) on five pairs of acrocentric chromosomes, silver (NOR) staining was performed. Briefly, fresh bone marrow or peripheral blood metaphases were prepared on clean glass slides and incubated with 50% AgNO_3_ solution plus formic acid-gelatin developer sandwiched between glass cover slips. The slides are heated to 70 °C for 3 to 4 min until the mixture turned yellow-brown and then they were rinsed with running water. Dark silver dots were deposited on the p arms of acrocentric chromosomes from the DRT patient and from normal controls. The silver deposits of 30 metaphases were scored and the mean number of Ag-NORs were calculated.

## Discussion and conclusions

RTs have been associated with various pre-malignant and malignant hematological disorders. Multiple acquired RTs have been reported in various leukemias [3, 5]. These include reports of Robertsonian translocation carriers who developed acquired leukemia (MDS/AML) later in life [5, 7]. One possible explanation for the anemia seen in our DRT patient is that he had a very limited number of NORs that were sufficient for ribosomal function in early life, however, aging led to further impairment/exhaustion of ribosomal function, resulting in anemia.

All human acrocentric chromosomes have nuclear organization regions (NORs) containing multiple copies of r-RNA genes located on the stalks of the short arms. There are ten acrocentric chromosomes in humans, but only seven to eight are active in dividing metaphases to generate Ag deposits in silver staining. When RT forms, NORs of the fusing acrocentric chromosomes are lost. Individuals with two double RTs have only six NORs instead of the usual ten, and more than 50% of NORs are inactive. Results for the current case study showed an average of only 3 to 4 Ag deposits on the normal acrocentric chromosomes (data not shown). This result shows a large reduction in the number of active NOR regions in this MA patient compared to normal healthy subjects.

With NOR reduction, there may be an insufficient quantity of NORs to affect normal DNA synthesis and cellular functions. The relationship between the reduced NOR quantity in this patient and the macrocytic anemia is undetermined. We hypothesize that the double RT may be etiologically responsible for the MA due to fewer NORs and subsequent reduced rRNA activity. Macrocytic anemia is characterized by the presence of abnormally large RBCs (mean corpuscular volume is greater than 100 fL) in the peripheral blood (mega blasts). Like other types of anemia, in macrocytic anemia, red blood cells may have low hemoglobin.

The most common causes of macrocytic anemias in adults are alcoholism, folate or vitamin B12 deficiency, hypothyroidism, liver diseases, hemolysis or bleeding, exposure to chemotherapy or certain drugs and myelodysplasia. Drugs that interfere with DNA synthesis, like hydroxyurea and azathioprine, are shown to cause macrocytosis with or without megaloblastic changes. There is also a benign familial form of macrocytosis. In some of the above conditions, macrocytosis is associated with abnormal nucleotide metabolism and/or defects in DNA polymerization [8].

Human ribosomopathies are a group of genetic disorders that are attributed to suboptimal ribosomal biogenesis or function due to specific mutations. Examples are Diamond-Blackfan anemia (a congenital bone marrow failure syndrome) and the 5q deletion syndrome--a myelodysplastic syndrome [9, 10]. Suboptimal ribosome function causes a severe macrocytic anemia in these diseases [10]. It is apparent that reduced availability of ribosomes can affect both DNA and protein synthesis. Ribosomal assembly needs a coordinated biogenesis precursor rRNA in nucleoli, transcription, modification and processing of rRNA. Ribosomal RNAs are synthesized in nucleoli which in humans are on short arms of all acrocentric chromosomes. A considerable reduction in the number of acrocentric p arms may reduce ribosomal RNA production and therefore reduce the biogenesis of functional ribosomes.

The underlying defect in macrocytosis is reduced DNA synthesis which can be due to the reduced number of active NORs. Delay in erythrocyte proliferation can be caused by abnormal DNA synthesis, nucleotide metabolism or DNA polymerization. Double RT formation resulted in the loss of four NORs containing actively transcribing rRNA genes. Substantial reduction in active NORs may impair DNA synthesis and result in macrocytic RBCs. Therefore, it is possible that the double RT present in this patient may be etiologically responsible for the MA due to fewer NORs and subsequently reduced rRNA activity.

To our knowledge, this is the first case report of a patient with macrocytic anemia harboring two heterologous RTs involving chromosomes 13 and 14 and chromosomes 14 and 15. Given the rarity of double RTs, it may not be possible to establish a direct correlation between reduced NOR regions and macrocytic anemia. Additional studies of patients with double RTs and anemia are required to further characterize such a relationship.

## Data Availability

Data from this case report is not available, due to privacy considerations. Data sharing is not applicable to this article, as no datasets were generated or analyzed during the current study.
